# Advanced outcomes of mixed reality usage in orthognathic surgery: a systematic review

**DOI:** 10.1186/s40902-024-00440-x

**Published:** 2024-07-29

**Authors:** Carolina Stevanie, Yossy Yoanita Ariestiana, Faqi Nurdiansyah Hendra, Muh Anshar, Paolo Boffano, Tymour Forouzanfar, Cortino Sukotjo, Sri Hastuti Kurniawan, Muhammad Ruslin

**Affiliations:** 1 Department of Oral and Maxillofacial Surgery, Faculty of Dentistry, Hasanuddin University, Makassar, Indonesia; 2 Department of Anatomy, Faculty of Medicine, Hasanuddin University, Makassar, Indonesia; 3 Department of Oral and Maxillofacial Surgery/ Oral Pathology, Amsterdam, UMC and Academic Centre for Dentistry Amsterdam (ACTA), Vrije Universiteit Amsterdam, Cancer Center Amsterdam, Amsterdam, The Netherlands; 4 Department of Electrical Engineering, Faculty of Engineering, Hasanuddin University, Makassar, Indonesia; 5 Department of Dentistry, AOU Maggiore Della Carità, Novara, Italy; 6 Department of Health Sciences, University of Eastern Piedmont, Novara, Italy; 7 Department of Oral and Maxillofacial Surgery, Leiden University Medical Center, Leiden, 2333 ZA Netherlands; 8 Department of Restorative Dentistry, University of Illinois Chicago College of Dentistry, Chicago, USA; 9 Department of Computational Media, Jack Baskin School of Engineering, University of California, Santa Cruz, USA

**Keywords:** Intraoperative navigation, Mixed reality, Orthognathic surgery, Virtual planning, Virtual training

## Abstract

**Introduction:**

Orthognathic surgery (OGS) is a highly sophisticated surgical technique that aims to repair a variety of skeletal and dental abnormalities, including misaligned jaws and teeth. It requires precise preoperative preparation and advanced surgical skills, which are typically learned through years of practical experience in operating rooms or laboratory-based surgical training facilities utilizing cadavers or models. The traditional physical hands-on method of surgical training is still used at OGS. However, this method requires a longer time of preparation. Currently, mixed reality (MR)—a combination of virtual reality and augmented reality technology—is an innovation of OGS. The present study aimed to present a comprehensive review of studies that assessed the advantages of utilizing mixed reality technology in OGS.

**Methods:**

A modified Population, Intervention, Comparison, Outcome strategy was performed using a combination of electronic (PubMed, Cochrane, Embase) and manual searches between 2013 and 2023 exploring mixed reality (MR) technology in OGS in the last 10 years. The inclusion criteria were limited to the patient and study model focusing on the clinical application of MR and the associated field of OGS.

**Result:**

The initial search indicated 1731 studies, of which 17 studies were included for analysis. The main results indicated that the use of MR technology in OGS led to high accuracy and time reduction as primary outcomes and cost-effectiveness and skill improvement as secondary outcomes. The review firmly concluded that MR technology exhibited a positive impact on students, trainees, and oromaxillofacial surgeons. However, due to the heterogeneity of the included studies, meta-analyses could not be performed. Collectively, these findings provide strong evidence for the advantages of MR technology in orthognathic surgery.

**Conclusion:**

MR technology significantly improves OGS planning efficiency by providing pre-surgical information and serving as an intraoperative navigation tool, reducing surgical time without compromising outcomes. Virtual training using MR technology exerts a positive impact on knowledge and skill improvement for OGS. This innovative technology will revolutionize the healthcare system and enhance patient care.

## Introduction

Orthognathic surgery (OGS), also called corrective jaw surgery, is the use of surgical procedures to correct imbalances in the upper and lower jaws [1, 2]. OGS enhances the soft tissues and bone structure of the face, resulting in improvements to both function and appearance [2, 3]. This procedure involves the conduct of osteotomies and realignment of the chin, mandible, and maxilla [2, 4].

A thorough assessment, precise planning, and professional understanding are necessary for this type of surgical management [2, 3, 5]. Surgeons accomplish extensive and comprehensive training to become familiar with these skills under the guidance of an experienced surgeon [6, 7]. However, due to several considerations, such as the effect on the patient’s comfort, the length of the surgical plan process, the time and cost of the surgery, and the probability of complications, this type of study approach is out of date [6–10].

Today, surgical simulators have been developed based on mixed reality (MR) technology [11, 12]. As MR consists of virtual reality (VR) and augmented reality (AR) technology, the relationship between both technologies relies on the use of virtual data as a 3D image to alter the physical world around the user, which allows surgeons to conduct correct surgical procedures, intraoperative tracking, and postoperative evaluation [13–16].

Recent studies have demonstrated the benefits of utilizing MR technology in various surgical procedures by improving the quality of the operation, accuracy, and precision, as well as shortened duration [17, 18]. However, the benefits of MR technology in OGS have not been sufficiently investigated [13, 19, 20]. The present systematic review aimed to provide a comprehensive analysis of the studies addressing MR technology utilization in OGS for the period between 2013 and 2023. In addition, attention is drawn to students, trainees, and oromaxillofacial surgeons about this technology to improve outcomes and shorten the duration of the operation.

## Materials and methods

### Review strategy and study registration

The present systematic review was accomplished and reported following the Preferred Reporting Items for Systematic Reviews and Meta-Analyses (PRISMA) guidelines [21]. The protocol for the systematic review was registered to PROSPERO (CRD42023455788).

### Focus question

The PICO framework for this investigation includes the following: the study population consisting of patients, animal, and study model receiving OGS/osteotomy procedure combined with MR (VR/AR) technology. The intervention examines OGS procedure on a study model or person with MR technology. Comparisons were made with alternative approaches for OGS between conventional OGS/osteotomy procedure and MR-based orthognathic procedure. Outcomes were assessed encompassing duration and accuracy as the primary outcome with cost-effective and knowledge improvement as secondary outcome as tabulated in Table 1.

**Table 1:** PICO framework

Population:	Patients, animals, and study models receiving OGS/ osteotomy procedure combined with MR (VR/AR) technology
Intervention:	OGS procedure (bimaxillary osteotomy, Le Fort I osteotomy, mandibular osteotomy, face analysis, cephalometric analysis, maxilla reposition, cutting and drilling, occlusion determination, etc.) on study model, animal or person with MR technology
Comparison:	Alternative approaches for OGS between conventional OGS/osteotomy procedure and MR-based orthognathic procedure
Outcome:	Duration and accuracy as primary outcome Cost-effective and knowledge improvement as secondary outcome

*Abbreviations*: Etc*.* et cetera, *OGS* Orthognathic surgery, *MR* Mixed reality, *VR* Virtual reality, *AR* Augmented reality

### Information sources and search approach

We performed an exhaustive search of electronic databases, including Pubmed, Cochrane, and Embase. The time frame for the published research articles is restricted to the last 10 years (2013–2023) to reflect the latest information. The initial search was carried out in December 2022 and updated in December 2023.

The article was limited to randomized clinical trial and clinical trial literatures using search terms such as “orthognathic surgery,” “mixed reality,” “augmented reality,” “virtual reality,” “surgical simulation,” “surgical planning accuracy,” “skill acquisition,” “conventional OGS,” and “training effectiveness.” Manual searches of notable journals related to MR technology utilization in OGS were also carried out. The search strategy was tailored for each specific database. A summary of the search strategies and the total number of studies retrieved are provided in Table 2.

**Table 2:** Custom search strategy of each database

Databases	Search strategy used	Hits
PubMed	(jaw surgery OR orthognathic surgery OR oral surgery AND mixed reality OR virtual reality OR augmented reality)	1679
Cochrane	#1 “orthognathic surgery OR maxillofacial surgery AND craniofacial deformities OR craniofacial anomalies” 5128 #2 “mixed reality OR augmented reality OR virtual reality OR 3D visualization OR surgical simulation” 1898 #3 “surgical planning accuracy OR training effectiveness OR navigation precision OR surgical proficiency OR learning curves OR skill acquisition” 28,541 #4 #1 AND #2 AND #3	19
Embase	orthognatic surgery AND mixed reality AND osteotomy OR conventional OGS	33

### Eligibility criteria and study selection

The eligible studies for inclusion in the present review were the following: (1) studies examining the outcomes of patients, animal, and study model receiving OGS/osteotomy procedure with MR technology, (2) analytical cross-section, cohort, case report, randomized controlled trial (RCT), or clinical trial study designs, and (3) human, animal, or study models as the study population.

Exclusion criteria were defined as follows: (1) review or systematic review articles, (2) research not involving MR technology in OGS/osteotomy procedure, and (3) not describing the advantage or outcome of the MR technology usage in OGS/osteotomy procedures.

Two review authors (C. S. and F. N.) independently screened the titles and abstracts of search results to identify relevant studies, considering the PICO question and the established inclusion and exclusion criteria. Irrelevant studies were excluded from the review, and the rationale for their exclusion was documented. In cases of disagreement between the authors, a third author (M. R.) was consulted for resolution. The full texts of potentially relevant articles were further evaluated, with those not adhering to the PICO framework or the inclusion and exclusion criteria being eliminated and reasons for their exclusion provided.

### Data items and collection process

One researcher (C. S.) extracted data from the selected articles, while another researcher (F. N.) confirmed the accuracy of the data extraction. Information of interest included the study author’s names, publication year, country of origin, the number of samples in treatment and the case, platform and devices, intervention, and outcomes such as duration/TCT, accuracy, cost-effective, and knowledge improvement. A summary of the data related to the duration/TCT of MR technology utilization in OGS is presented in Table 3, while a summary of the accuracy obtained of MR technology utilization in OGS is presented in Table 4.

**Table 3:** Duration/TCT analysis results based on intervention for MR technology utilization in OGS

Author, country	Year	Number of subjects, case	Platform, devices	Duration/TCT								
				**FA**	**CA**	**BMO**	**LF1**	**SO**	**M**	**SJ**	**DJ**	**MR**
Zinser et al. [22] Germany	2013	16 patients, Skeletal class III malocclusion combined with open bite or vertical maxillary extrusion	BrainLab®, Vector vision	N/S	N/S	288 min	N/S	N/S	N/S	N/S	N/S	N/S
Badiali et al. [23] Italy	2015	15 patients, Class II or Class III dentofacial deformities with severe asymmetry and complex vertical dimension (short/long face)	- WARM (Wearable Augmented Reality for Medicine) - Z800 eMagin (Bellevue, WA, USA)	N/S	N/S	N/S	180–240 min	N/S	N/S	N/S	N/S	5–10 min
Lutz et al. [24] France	2015	1 3D-printed model	FW4SPL open source, Virtual kit library	N/S	N/S	N/S	N/S	N/S	N/S	N/S	N/S	41.87 s (exp.) 30.88 s (tr.)
Steinhuber et al. [25] Austria	2018	40 patients, Skeletal class II and class III profile	Dolphin Imaging Software version 11.9; Dolphin Imaging and Management Solutions, Chatsworth, CA, USA	N/S	N/S	N/S	N/S	N/S	N/S	41.2 min (surg.) 22.8 min (assist.) 45.3 min (tech.)	53.6 min (surg.) 20.9 min (assist.) 75.1 min (tech.)	N/S
Gao et al. [26] China	2019	1 dental model, Mandibular angle prominence	- Unity 3D engine (Microsoft Corporation, Redmond, WA, USA) - Vuforia (PTC Inc.,USA)	N/S	N/S	N/S	N/S	6.45–8.81 min. (surg.) 6.26–9.22 min. (eng.)	N/S	N/S	N/S	N/S
Zaragoza et al. [27] Mexico	2019	-Patient’s frontal and lateral photograph (module I) -Lateral photograph (module II) -3D Models from CT-Scan (Module III and IV) - 25 years old male 3D model (Module V)	- OSSys System - Omni phantom (Sensable®) - Falcon (Novit®)	5.5 min (nov.) 3.2 min (exp.)	7.5 min (nov.) 8.2 min (exp.)	N/S	N/S	- 7.5 min (nov.) - 8.2 min (exp.)	N/S	N/S	N/S	N/S
Sakowitz et al. [16] USA	2019	3 cases: - Case 1 for trial - Case 2 simple discrepancy - Case 3 discrepancy of both jaws	- Oculus Rift DK2 (Oculus - VR, Menlo Park, CA)	N/S	Case 2: 15.05 min Case 3: 15.5 min	N/S	N/S	N/S	N/S	N/S	N/S	N/S
Medellin et al. [28] Mexico	2020	5 patients with malocclusion One 3D cephalometry	- OSSys System - Omni phantom (Sensable®) -Falcon (Novit®)	N/S	< 2 min (nov.)	N/S	N/S	53 sec. (V) 21.5 sec. (VH)	19.4 s. (V) 11.6 s. (VH)	N/S	N/S	N/S

*Abbreviations*: *FA* facial analysis, *CA* cephalometric analysis, *BMO* bimaxillary osteotomy, *LF1* Le Fort 1, *SO* sagittal osteotomy, *M* mentoplasty, *SJ* single jaw, *DJ* double jaw, *Nov* novices, *Exp* expert, *Surg* surgeon, *Assist* assistant, *Eng* engineer, *V* virtual only, *VH* virtual and haptic enabled, *Mins* minutes, *Sec* seconds, *N/S* not specified

**Table 4:** Accuracy analysis results for MR technology utilization in OGS

Author, country	Year	Number of subjects, case	Platform, devices	Accuracy parameters					
				**PE**	**L/DE**	**AE**	**CE**	**TE**	**OE**
Zinser et al. [22] Germany	2013	16 patients, Skeletal class III malocclusion combined with open bite or vertical maxillary extrusion	BrainLab®, Vector vision	N/S	ML-MP (0.01–0.2 mm ML-CP (0.19–0.37 mm) VD-FHP-FZL (0.12–0.37 mm)	(OP-FHP) < 0.35°	N/S	N/S	N/S
Badiali et al. [23] Italy	2015	15 patients, Class II or Class III dentofacial deformities with severe asymmetry and complex vertical dimension (short/long face)	- WARM (Wearable Augmented Reality for Medicine) - Z800 eMagin (Bellevue, WA, USA)	N/S	XYZ Direction: - Frontal: 1.91 mm - Caudal-cranial: 0.59 mm - Lateral: 1.02 mm	N/S	N/S	N/S	N/S
Lutz et al. [24] France	2015	1 3D-printed model	- FW4SPL open source, Virtual kit library - VR-Med Software	N/S	N/S	1.32°	N/S	1.11 mm	N/S
Fushima et al. [11] Japan	2016	3D craniofacial dental model	ManMos (Mandibular motion tracking system)	N/S	< 0.32 mm	N/S	N/S	N/S	N/S
Ricciardi et al. [29] Italy	2017	- 4 3D-printed models - 1 frontal half-model	- IGSTK (Image Guided Surgery Toolkit) - VTK Framework (mesh editor) - Qt Framework (application interface) - NDI Polaris Vicra optical tracker - Philips SPC1330NC webcam	N/S	- 1.37 mm (2 anatomical points, 3 screws) - 1.43 mm (5 anatomical points)	N/S	N/S	N/S	N/S
Wu et al. [30] China	2017	15 sets of dental models	GPU-based collision detection method	N/S	- X, 0.022–0.648 mm - Y, 0.047–0.371 mm - Z, 0.019–1.139 mm	N/S	N/S	N/S	N/S
Gao et al. [26] China	2019	1 dental model, Mandibular angle prominence	- Unity 3D engine (Microsoft Corporation, Redmond, WA, USA) - Vuforia (PTC Inc.,USA)	1.36–3.22 mm (eng.) 1.38–4.43 mm (surg.)	N/S	N/S	N/S	N/S	- 0.88–3.18 mm (eng.) - 0.88–3.18 mm (surg.)
Ahn, et al. [31] Korea	2019	1 patient	Visual C + + (Microsoft, Redmond, WA)	0.0596 mm	N/S	N/S	N/S	N/S	N/S
Medellin et al. [28] Mexico	2020	- 5 patients with malocclusion - 1 3D cephalometry	- OSSys System - Omni phantom (Sensable®) - Falcon (Novit®)	N/S	N/S	N/S	Mentoplasty: - V, 14.6% - VH, 6.4% Sagittal osteotomy: - V, 21.2% - VH, 4.9%	N/S	N/S
Koyachi et al. [32] Korea	2021	18 patients	Microsoft Hololens	N/S	- 2 mm (accuracy < 90% in 12 cases) - XYZ deviation, 0.38 mm	N/S	N/S	N/S	N/S
Jo, et al. [33] Korea	2021	1 patient	- Unity 3D engine (Microsoft Corporation, Redmond, WA, USA) - Mimics software (18.0, Materialise, Leuven, Belgium) - Vive pro (HTC Vive, Taipei, Taiwan) - HMD (Head mounted display)	N/S	3 mm (SD, 1.44 mm)	N/S	N/S	N/S	N/S

*Abbreviations*: *PE* position error, *L/DE* linear/distance error, *AE* angular error, *CE* cutting error, *TE* translation error, *Surg* surgeon, *Eng* engineer, *V* virtual only, *VH* virtual and haptic enabled, *SD* standard deviation, *ML* maxillary landmark, *OP* occlusal plane, *MP* midfacial plane, *CP* coronal plane, *VD* vertical dimension, *FHP* Frankfurt horizontal plane, *FZL* frontozygomatic line, *N/S* not specified

### Assessing risk of bias

All studies included in the present systematic review consisted of 4 types of study designs, namely cross-sectional [22, 34–36], case report [33], prospective cohort [25], and randomized controlled trial [11, 16, 23, 24, 26–32]. We evaluated their risk of bias using Joanna Briggs Institute (JBI) assessment tool based on the type of study design of studies involved. The results of this assessment are tabulated in Table 5.

**Table 5:** The risk of bias of included studies based on the Joanna Briggs Institute (JBI) assessment

JBI assessment tools	Questions													Score % (category of bias)
**Cross-sectional** [37]	**Q1**	**Q2**	**Q3**	**Q4**	**Q5**	**Q6**	**Q7**	**Q8**						
Zinser 2013 [22]	✓	?	✓	✓	✕	✓	✓	✓						75
Pulijala 2018 [34]	✓	✓	✓	✓	✕	✕	✓	✓						75
Arikatla 2018 [35]	✓	✓	✓	✓	✕	✕	✓	?						62.5
Pham Dang 2021 [36]	✓	✓	✓	✓	?	?	✓	✕						62.5
**Average score**														**68.75 (moderate risk)**
**Cohort** [38]	**Q1**	**Q2**	**Q3**	**Q4**	**Q5**	**Q6**	**Q7**	**Q8**	**Q9**	**Q10**	**Q11**			
Steinhuber 2018 [25]	✓	✓	✓	✓	?	?	✓	✓	✓	✓	✓			**81 (low risk)**
**Case report** [38]	**Q1**	**Q2**	**Q3**	**Q4**	**Q5**	**Q6**	**Q7**	**Q8**						
J o 2021 [33]	✓	?	✓	✓	✓	✓	✓	✓						**87.5 (low risk)**
**RCT** [39]	**Q1**	**Q2**	**Q3**	**Q4**	**Q5**	**Q6**	**Q7**	**Q8**	**Q9**	**Q10**	**Q11**	**Q12**	**Q13**	
Lutz 2015 [40]	✓	✓	✓	✓	✓	✓	✓	✓	✓	✓	✓	✓	✓	100
Badiali 2015 [23]	✓	✓	✓	✓	✓	✓	✓	✓	✓	✓	✓	✓	✓	100
Fushima 2016 [11]	✓	✓	✓	✓	✓	✓	✓	✓	✓	✓	✓	✓	✓	100
Wu 2017 [30]	?	✓	✓	✓	✓	✓	✓	?	✓	✓	✓	✓	✓	84.6
Ricciardi 2017 [29]	✓	✓	✓	✓	✓	✓	✓	✓	✓	✓	✓	?	✓	92.3
Zaragoza 2019 [27]	✓	✓	✓	✓	✓	✓	✓	✓	✓	✓	✓	✕	✓	84.6
Sakowitz 2019 [16]	✓	✓	✓	✓	✓	✓	✓	✓	✓	✓	✓	?	✓	84.6
Gao 2019 [26]	✓	✓	✓	✓	✓	✓	✓	✓	✓	✓	✓	✓	✓	100
Ahn 2019 [31]	✓	✓	✓	✓	✓	✓	✓	✓	✓	✓	✓	✓	✓	100
Medellin 2020 [28]	✓	✓	✓	✓	✓	✓	✓	✓	✓	✓	✓	✓	✓	100
Koyachi 2021 [32]	✓	✓	✓	✓	✓	✓	✓	✓	✓	✓	✓	✓	✓	100
**Average score**														**95.5 (low risk)**

*Abbreviations*: ✓ yes, ✕ no, ? unclear, ⦸ not applicable

### Synthesis of the summary measures

The data obtained from the selected articles were deemed appropriate as the primary outcome if they showed the effectiveness of MR technology usage in OGS/osteotomy procedure in terms of duration/TCT and accuracy. Other findings, such as cost-effectiveness and knowledge improvement, were considered secondary outcomes. Validated comparisons among selected publications were not feasible due to heterogeneity present in the field of OGS and MR technology, and meta-analyses were not accomplished.

The data was assembled using the following parameters and subsequently organized according to predefined schemes as mentioned below:

- “Author, country”—It was used to reveal the first author and country where the study was conducted.
- “Year” —described the year of publication of literature.
- “Number of subjects” —It was used to describe the type and size of samples used.
- “Case”—It was used to describe the condition of maxillofacial abnormalities which need OGS procedure.
- “Platform, devices”—It was used to explain which platforms and devices are used in literature.
- “Accuracy”—It was used to show the deviation between virtual intervention and actual intervention performed in the research.
- “Duration/Time completion task (TCT)”—It was used to describe the duration required for completing the training task/the operation.
- “Knowledge improvement”—It was used to describe the measurement indicators used in assessing the knowledge of virtual training participants.
- “Cost-effective”—it was used to describe the reducement of production cost.

## Result

### Study selection

After eliminating duplicate entries, 1747 articles were identified through the search approach. A thorough assessment of titles and abstracts led to the exclusion of articles, leaving 84 articles with potential relevance.

The 84 full-text articles from databases underwent a screening process based on predeterminated inclusion and exclusion parameters. Upon examining the reference lists of these articles, 29 more studies were added. In the end, seventeen studies met the criteria and were incorporated into the review, while 38 were dismissed after a full-text evaluation.

**Fig. 1:**
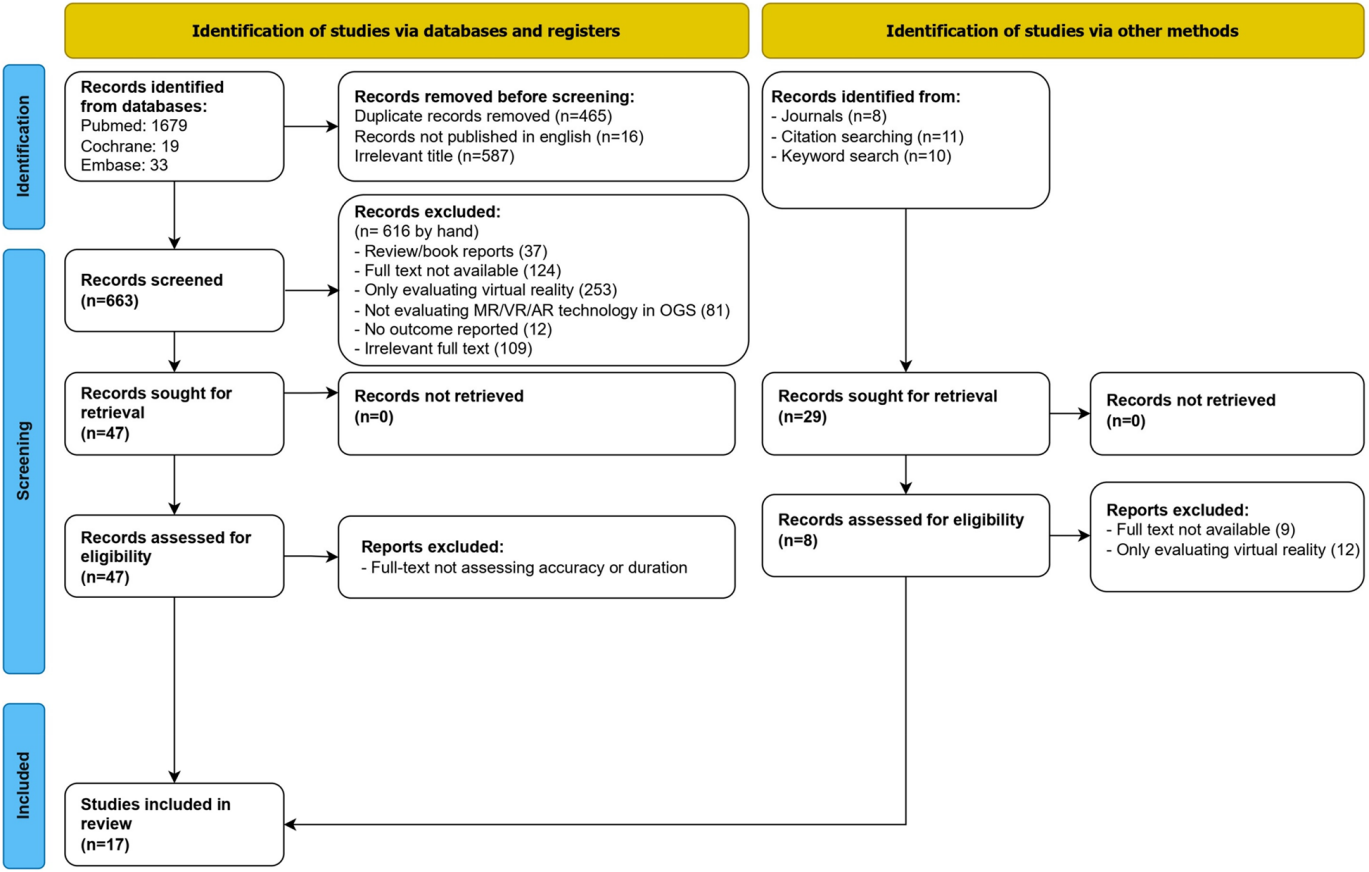
Flow chart of the article selection process

A flowchart depicting the process of identifying, including, and excluding studies along with the reasons for exclusion is presented in Fig. 1.

### Study characteristics

**Fig. 2:**
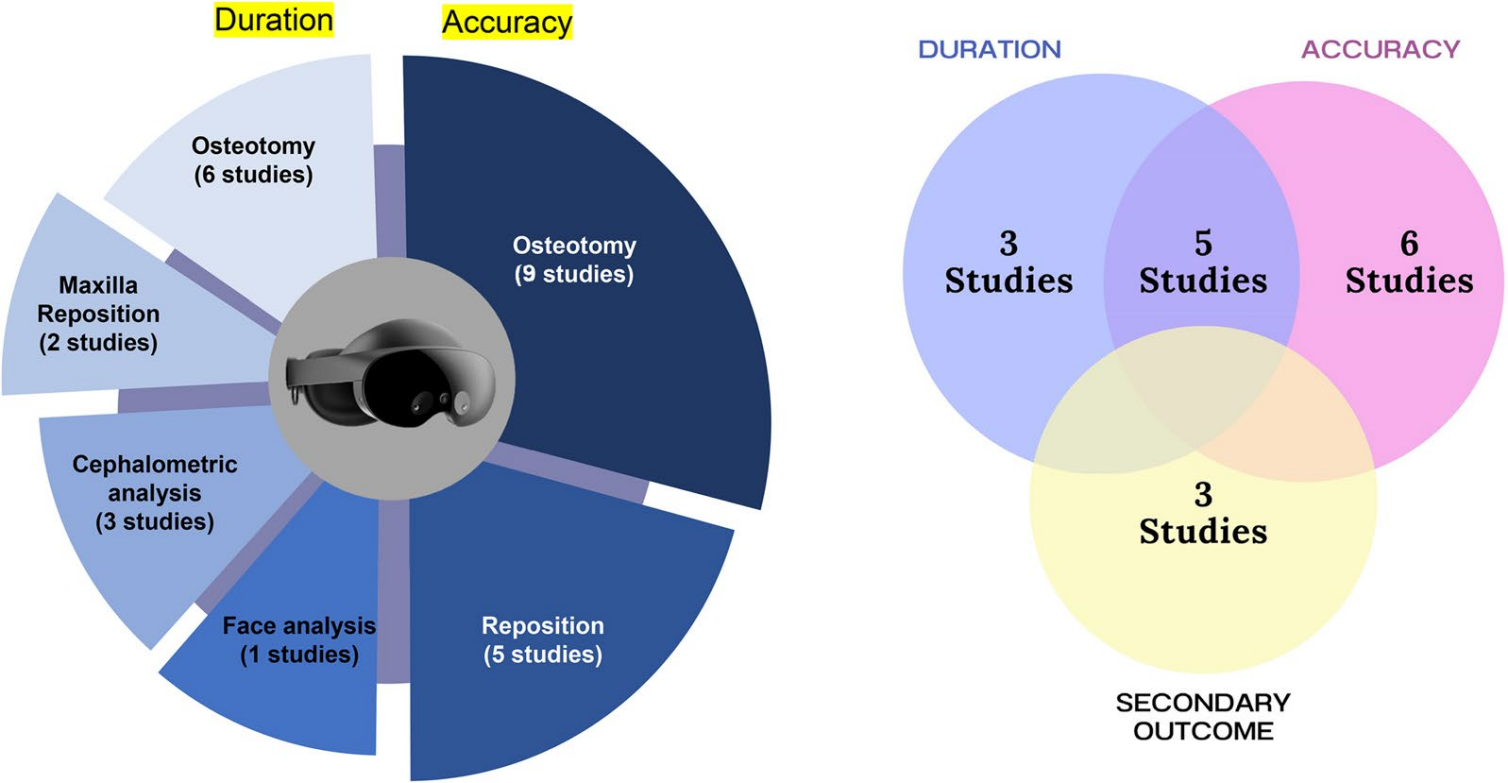
Intervention characteristics for duration and accuracy outcome. Left: studies distribution based on intervention. Right: studies distribution based on outcome

The systematic review encompassed seventeen studies, with four being cross-sectional [22, 34–36], one case report [33], one prospective cohort [41], and eleven randomized controlled trials [11, 16, 23, 24, 26–32, 40] (Fig. 2). These studies took place between 2013 and 2023, with patients involved in ten studies [22, 23, 25, 27, 28, 31–33, 35, 36], animals involved in one study [36], and dental study models involved in five studies [16, 26, 27, 30, 36]. Surgical planning was evaluated in six studies which focused on preoperative maxillary repositioning, occlusion determination, osteotomy plan, cutting, and drilling [16, 22, 25, 27, 30, 36]. The effectiveness of MR technology as an intraoperative navigator was evaluated in six studies [11, 23, 26, 31–33]. The intervention was performed using real-time 3D visualization for maxillary reposition, Le Fort I osteotomy, intermediate splint, bimaxillary osteotomy, and mandibular osteotomy.

The remaining five studies demonstrated the effectiveness of MR technology as a training tool for students, trainees, and oromaxillofacial surgeons to escalate their understanding regarding OGS [6, 7]. The intervention performed on the simulator included face analysis, cephalometry analysis drilling, cutting, oscillating saw usage, osteotomy Le Fort I, mandibular osteotomy, and case-based training. These studies showed the improvement of technical skills and performance between novices and experienced surgeons, as well as the reduction in duration/TCT of OGS procedure.

Among the included studies, the most commonly used software was Dolphin [25], Mimics [33], ManMos [11], VR-Med [33], and Unity 3D engine [26, 33]. The summary of the duration/TCT analysis is presented in Table 3, and the summary of accuracy is presented in Table 4.

### Risk of bias

Upon evaluating the seventeen studies using the JBI assessment tool, the risk of bias was found to be diverse, with final assessment scores ranging from 68% (moderate risk) [22, 34–36] to 100% (low risk) [11, 16, 23–33]. The study exhibits a mix of methodological which should be assessed based on the study designs for interpreting the results.

Out of seventeen studies, five studies included the control group [24–28], facilitating more robust comparisons and outcome evaluations. In contrast, the remaining twelve studies without control groups had limited generalizability and introduced bias into their results [11, 16, 22, 23, 29–36]. The risk of bias assessment of all the studies can be found in Table 5.

### Summary of results

In the studies reviewed, various assessments were conducted to evaluate the benefits of MR technology in OGS procedure as planning, intraoperative navigation, and pre-surgical training tools by assessing the accuracy, duration/TCT, cost-effective, and knowledge improvements.

Out of the seventeen studies that met the criteria, five studies described the advantages of MR technology about accuracy and TCT/duration [22, 23, 26, 28, 40], three studies explained the advantages about TCT/duration only [16, 25, 27] and six studies described the advantages of MR technology in terms of accuracy only [11, 29–33], while the remaining studies explained the advantages of MR in cost-effective and knowledge improvement [34–36].

### Accuracy analysis result

The accuracy defines how close a measurement is to the actual value by comparing the position error (PE), linear/distance error (L/DE), angular error (AE), cutting error (CE), translation error (TE), and orientation error (OE), the accuracy may be evaluated [42]. Gao et al. [26] reported that the position error of the cutting plan and osteotomy by the surgeon was 1.38–4.43 mm, while the engineer position error hits 1.36–3.22 mm. The orientation error was reported similarly between surgeon and engineer (0.88–3.18 mm). In line with that, Ahn et al. [31] indicated a low position error of maxilla reposition, with a mean error of 0.0596 mm.

Distance error was evaluated by several studies to promote the accuracy of MR technology. Koyachi et al. [32] described *X*-axis as the middle point that passes between the orbitales on both sides, the *Y*-axis as the vertical cranial direction from the Frankfort horizontal plane, and the *Z*-axis as the right-hand direction from the center. Zinser et al. [22] observed remarkable accuracy of bimaxillary osteotomy on 16 malocclusion skeletal class III patients with distance error between maxillary landmark to mandibular plane (0.01–0.2 mm), maxillary landmark to coronal plane (0.19–0.37 mm), and vertical dimension to Frankfurt horizontal plane (FHP) to frontozygomatic line (0.12–0.37 mm) with the acceptable angular error between occlusal plane and FHP (< 0.35°).

Ricciardi et al. [29] reveal that MR technology utilization for reconstruction planning and drilling guidance has low distance error and varies between 1.37 and 1.43 mm. Studies by Koyachi et al. [32] and Jo et al. [33] also found low distance error on osteotomy Le Fort I and maxillary reposition, with XYZ deviation 0.38 mm, accuracy of more than 90%, and 2–3 mm distance error. Similarly, Fushima et al. [11] found distance error of mandibular osteotomy approximately < 0.32 mm.

In XYZ direction, Badialli et al. [23] show low distance error on virtual planned osteotomy and maxilla-mandible reposition, with deviation on frontal (1.91 mm), caudal-cranial (0.59 mm), and lateral (1.02 mm). Similarly, Wu et al. [30] found distance error in *X* direction (0.022–0.648 mm), *Y* direction (0.047–0.371 mm), and *Z* direction (0.019–1.139 mm).

Medellin et al. [28] evaluated cutting error after virtual training with and without haptic for mentoplasty and sagittal osteotomy. The use of virtual training only shows cutting error of mentoplasty within 14.6% and sagittal osteotomy within 21.2%. This value was significantly reduced with the combination of haptic-enabled device. The cutting error was reduced to 6.4% for mentoplasty and 4.9% for sagittal osteotomy. 

Translation error is defined as the Euclidean distance between the centers of source and target [42]. Lutz et al. [24] utilized VR-Med software and electromagnetic (EM) tracking system for maxilla reposition with translation error within 1.11 mm.

In response to this research, MR technology has proven to be a valuable asset in orthognathic surgery practice, with excellent precision and accuracy. Through seamlessly merging virtual aspects with the actual world, it improves spatial awareness and offers surgeons with comprehensive anatomical visualization. This exact integration improves preoperative planning and provides surgeons with real-time data throughout treatments, providing more predictable and successful outcomes for patients undergoing orthognathic surgery.

### Duration/TCT analysis result

TCT is described as the reducement of time or duration needed for surgeons to complete the OGS procedure with the utilization of MR technology [28]. Face analysis and cephalometric analysis are considered important steps in OGS planning. Zaragoza et al. [27] evaluated five modules of OGS procedure, consisting of face analysis, cephalometric analysis, surgical template, model surgery, and case-based study. According to the study, the face analysis performed by novices and experts takes almost the same amount of time, 5.5 min and 3.2 min respectively. Similarly, the cephalometric analysis performed by novices and experts had slightly different durations of 7.5 min and 8.2 min, respectively [27].

Another study by Medellin et al. [28] reported cephalometric analysis performed by novices using OSSys system reduced the TCT by less than 2 min. In line with that, Sakowitz et al. [16] evaluated cephalometric analysis in 3 cases (trial case, simple discrepancy, and discrepancy on both jaws) utilizing VR technology. The duration required to solve cases 2 and 3 is relatively fast, with 15.05 min and 15.5 min, respectively.

Medellin et al. [28] also evaluated the use of virtual and haptic device on sagittal osteotomy and mentoplasty procedure. It reveals that the TCT reduced when combining virtual and haptic-enabled device, which was 21.5 s for sagittal osteotomy and 11.6 s for mentoplasty, while the use of virtual only needs longer duration, which was 53 s for sagittal osteotomy and 19.4 s for mentoplasty [28]. In line with that, Gao et al. [26] compared the TCT of sagittal osteotomy between surgeon and engineer and found that there are slight differences. The surgeon needs 6.45–8.81 min, while the engineer needs 6.26–9.22 min to complete the procedure. In accordance, Badiali et al. [23] discovered lower TCT on Le Fort I osteotomy, which was 180–240 min.

Steinhuber et al. [25] evaluated the use of virtual surgical planning (VSP) in the management of single-jaw and double-jaw osteotomies performed by surgeon, assistant, and technician. They discovered that the duration of single-jaw surgery performed by an assistant was lower than surgeon and technician, which was 22.8 min, while the surgeon and technician were 41.2 min and 45.3 min, respectively. Similarly, in the case of double-jaw surgery, the duration needed by the assistant was lower than surgeon and technician, which was 20.9 min, while the surgeon and technician were 53.6 min and 75.1 min, respectively [25].

Lutz et al. [24] discovered the reduced time for maxillary reposition by comparing experts and trainees. Trainees perform the maxillary reposition 10.99 s faster than experts. In another study, Lutz et al. [40] developed a semi-automated segmentation pipeline using VR-Med software. Concerning accuracy, the mean distance error range was 0.72–1.68 mm, with the number of outliers not exceeding 25%. The accuracy of the simulation model was < 1 mm, providing realistic simulation, as expected from the comparison with clinical images. Navigation significantly improved accuracy and operative time for less experienced surgeons, notably in complex bone movements along multiple axes [40].

Medellin et al. [28] and Zaragoza et al. [27] demonstrated the use of MR technology as a tool for clinical learning, facial analysis, and cephalometry, training in managing sagittal osteotomies and mentoplasty, and creating surgical templates. Compared with traditional techniques, MR technology can reduce the whole procedure time to 240.9 min (± 4 h) for experienced surgeons and 456.6 min (± 7 h) for novice surgeons.

All of this study reinforces that MR technology helps reduce the skill gap between surgeons and novices. The skill gap helps surgeons improve surgical outcomes by performing correct procedures and minimizing surgical complications.

### Knowledge improvements

Education centers are encouraged to explore innovative methods that complement traditional training for understanding OGS. Cost-effective and knowledge improvement were reported in several studies as the benefits of MR, enabling students to explore the anatomy and identify anomalies in a virtual environment and positively impacts the training method of OGS, increasing student engagement in the subject so that information can be distributed effectively and motor skills can be improved.

Pulijala et al. [43] reported that first year residents exhibited a significantly higher level of confidence in performing Le Fort I osteotomy in comparison to those in the second and third years. In another study, Pulijala et al. [9] evaluate the validity of virtual reality surgery using a questionnaire. The result indicated that the VR-based training tool developed exhibited a satisfactory level of validity. It can be used among surgical trainees in oral and maxillofacial surgery to enhance their non-surgical skills and improve their knowledge of OGS.

Pham Dang et al. [11] utilized MR technology to identify significant structures that are not clinically visible during surgery. MR technology accurately identified the human dental cusp, as well as the two mental foramina of the porcine head and the inferior alveolar nerve on phantoms. It is believed that this will aid surgeons in anticipating the challenges they will face during surgery to reduce the risk of complications. Similarly, Arikatla et al. [35] develop complicated OGS techniques that offer accurate simulation for a crucial aspect of the treatment, particularly real-time bone cutting by utilizing MR technology.

Based on a thorough review of these studies, it is recommended that MR technology supports the improvement of skills and knowledge of students, trainees, and orthomaxillofacial surgeons by enabling real-time OGS training to complement educational methods. This innovation encourages students, trainees, and orthomaxillofacial surgeons to explore more about OGS and possible complications.

### Cost-effective

Despite the numerous benefits of using MR technology, its use is limited in developing nations due to its costly production and the investment required for accurate surgical simulators [44]. Bengtsson et al. [45] compared the financial costs for radiographic examination and surgical planning between VSP and TSP method. Time spent is converted to financial cost using the economic cost/unit of time according to the local average salary of an oral and maxillofacial surgeon. The cost of the initial investment was higher for VSP ($16,471) than TSP ($2588). Abbate et al. [46] estimated a cost of approximately 6.3 Euros per case. While it may require a significant initial investment, the long-term benefits of using MR technology can outweigh the costs, notably for countries with limited resources.

However, there is a growing interest in analyzing the cost–benefit of using MR technology for routine surgery, as it may be a worthwhile investment in the future compared with 3D models [47, 48]. This is in line with research conducted by Sutherland et al. [49] that use two smartphones and a VR headset for slit lamp and surgical videos in 3D view. This demonstrates that MR technology can provide in a simpler and low-cost component.

All of these included studies underscore the potential benefits of employing MR technology as an investment for the development of the field of education, especially oral and maxillofacial surgery. Lectures can utilize this technology continuously and can be developed in more in-depth learning modules. Case scenarios allow students to learn and prepare themselves before performing the actual surgery.

## Discussion

The present systematic review focuses on clinical research exploring the application of MR technology in OGS. Clinical studies of OGS that use MR technologies are mainly experimental and are utilized for science projects or educational purposes that follow specific surgical protocols. The latest computing hardware and supporting software are essential for enabling the use of MR in healthcare activities. MR technology has a proven track record of effectively reducing planning time with optimal reliability.

All of the seventeen studies that were analyzed in the present systematic review indicated that MR technology was highly accurate in OGS planning as well as during the actual procedure. Following a thorough review, it was found that every publication demonstrated minimal deviation between predicted and actual outcomes for repositioning and osteotomy surgeries. The present study is consistent with the findings of Ayoub et al. [13], who discovered the accuracy of virtual planning for orthognathic surgery. According to their evaluation, virtual planning is a reliable method that aids the majority of the clinicians to reduce preparation time.

Furthermore, a study done by Wilkat et al. [12] developed a prototype tool for virtual occlusion and compared it with the conventional method. It concluded that MR technology helps reduce the material cost and personal effort, leading to less operation time and providing interactive and highly informative platform. Similarly, Koyachi et al. [50] reported that the overlay error of genioplasty procedure between the operative virtual operation and 1-month postoperative CT data within 2 mm was 100%, by utilizing the combination of computer-aided design and computer-aided manufacturing (CAD/CAM) with MR technology. It shows that the combination of CAD/CAM and MR technology enabled accurate planning for genioplasty.

Another systematic review was done by Chen et al. [51] evaluating randomized clinical trials about VSP and TSP and concluded that VSP shortened the planning and operating time, giving the same satisfaction to the patient’s post operative outcome, and the cost-effective between conventional and virtual surgical plan remain similar considering the time production and printed model costs.

Although only seventeen studies were included in the present systematic review, the increasing number of published articles per year indicates the high public interest in using MR in the healthcare field. MR technology has both potential and limitations, specifically regarding technical performance, sensor tracking, and clinical use regulation [52, 53]. According to FDA regulations [54], MR technology is allowed for clinical use in the medical field to promote safety and effectiveness of healthcare as well as increase patient’s compliance and adherence to the therapy. A specialized training is considered for clinicians to utilize MR technology safely and avoid malfunction. Developed MR technology should be registered to the FDA’s medical reporting tool, MedWatch, before it can be widely distributed. Similarly, John et al. [19] found that MR technology in the medical field can enhance the effectiveness of medical education and training, raise the level of diagnosis and treatment, improve the doctor-patient relationship, and boost the efficiency of medical implementation.

### Limitations and suggestions for further research

High heterogeneity in the included publications was observed, resulting in comparisons, validation, and meta-analysis not being possible. Standardized parameters for evaluating surgical simulators are required to enable fairer comparisons and focus on important aspects for the targeted application.

## Conclusion

MR technology has provided benefits to OGS education and practice. Students, trainees, and oromaxillofacial surgeons are expected to use the technical skills they learn in virtual surgical simulators to prepare their minds and bodies for actual surgery. Pre-surgical training can help reduce the stressful operating theater environment and the difficulty of surgery and anticipate difficult circumstances. As a result, the skill gap between novice surgeons and experienced surgeons can be reduced. The importance of this breakthrough in improving patient care standards has been mentioned in most papers. In the near future, we will see the full potential of MR technology in the field of oral and maxillofacial surgery as a result of ongoing technological developments.

## Data Availability

This is a review article. All data are taken from published research papers and available online.

## Abbreviations

M.R: Muhammad Ruslin
F.N: Faqi Nurdiansyah Hendra
C.S: Carolina Stevanie
OGS: Orthognathic surgery
PICO: Population, Intervention, Comparison, Outcome
MR: Mixed reality
AR: Augmented reality
VR: Virtual reality
2D: Two dimensional
3D: Three dimensional
VSP: Virtual surgical planning
TSP: Traditional surgical planning
PRISMA: Preferred Reporting Items for Systematic Reviews and Meta-Analyses
TCT: Time completion task
JBI: Joanna Briggs Institute

## References

[1] Ruslin M, Forouzanfar T, Astuti IA, Soemantri ES, Tunzing DB (2015) The epidemiology, treatment, and complication of dentofacial deformities in an Indonesian population: a 21-year analysis. J Oral Maxillofac Surg Med Pathol 27(5):601–607 10.1016/j.ajoms.2014.09.006

[2] Choi JW, Lee JY. (2021) Surgical treatment objectives and the clinical procedure for the surgery-first approach. In: The surgery-first orthognathic approach. Springer: 21–36.

[3] Naini FB, Gill DS. (2017) Principles of orthognathic treatment planning. In: Orthognathic surgery. Wiley Blackwell: 170–213.

[4] Eslamipour F, Farahani, AB., Le BT, Shahmoradi M. (2017) A retrospective Analysis of Dentofacial Deformities and Orthognathic Surgeries. Ann Maxillofac Surg 7(1). 10.4103/ams.ams_104_16 PMC5502519 28713739

[5] Shetty SK, Neeraja Yethadka MK, Vivek V (2017) CBCT in orthognathic surgery. Sch J Dent Sci 4(12):547–555

[6] Reyneke JP, Ferretti C. (2021) Diagnosis and planning in orthognathic surgery. In: Oral and maxillofacial surgery for the clinician, Springer: 1437–1462.

[7] Sousa CS, Turrini RNT (2019) Development of an educational mobile application for patients submitted to orthognathic surgery. Rev Lat Am Enfermagem 27:1–9 10.1590/1518-8345.2904.3143 PMC6687363 31340340

[8] O’Malley AM, Milosevic A (2000) Comparison of three facebow/semi-adjustable articulator systems for planning orthognathic surgery. Br J Oral Maxillofac Surg 38(3):185–190 10864723 10.1054/bjom.1999.0182

[9] Ho CT, Lin HH, Lo LJ (2019) Intraoral scanning and setting up the digital final occlusion in three-dimensional planning of orthognathic surgery: its comparison with the dental model approach. Plast Reconstr Surg 143(5):1027e–1036e 31033828 10.1097/PRS.0000000000005556

[10] Seo HJ, Choi YK (2021) Current trends in orthognathic surgery. Arch Craniofac Surg 22(6):287–295 34974683 10.7181/acfs.2021.00598 PMC8721433

[11] Fushima K, Kobayashi M (2016) Mixed-reality simulation for orthognathic surgery. Maxillofac Plast Reconstr Surg 38(13):1–12 27014664 10.1186/s40902-016-0059-z PMC4783436

[12] Wilkat M, Liu S, Schwerter M, Schrader F, Saigo L, Karnatz N et al (2023) A new approach to virtual occlusion in orthognathic surgery planning using mixed reality-a technical note and review of the literature. J Pers Med 13:1–18 10.3390/jpm13121709 PMC10744857 38138936

[13] Ayoub A, Pulijala Y (2019) The application of virtual reality and augmented reality in oral & maxillofacial surgery. BMC Oral Health 19(238):1–8 31703708 10.1186/s12903-019-0937-8 PMC6839223

[14] Liao YF, Chen YA, Chen YC, Chen YR (2020) Outcomes of conventional versus virtual surgical planning of orthognathic surgery using surgery-first approach for class III asymmetry. Clin Oral Investig 24(4):1509–1516 32100114 10.1007/s00784-020-03241-4

[15] Lonic D, Lo LJ. Three-dimensional simulation of orthognathic surgery-surgeon’s perspective. J Formosan Med Assoc 115(6):387–388. 10.1016/j.jfma.2015.09.002 26482093

[16] Sakowitz SM, Inglehart MR, Ramaswamy V, Edwards S, Shoukri B, Sachs S, Kim-Berman H (2020) A comparison of two-dimensional prediction tracing and a virtual reality patient method for diagnosis and treatment planning of orthognathic cases in dental students: a randomized preliminary study. Virtual Reality 24(3):399–409 10.1007/s10055-019-00413-w

[17] Dougls DB, Wilke CA, Gibson D, Petricoin EF, Liotta L. (2017) Petricoin, and L. Liotta. (2017) Virtual reality and augmented reality: advances in surgery. Biol Eng Med 3(1).

[18] Moro A, Pelo S, Gasparini G, Saponaro G, Todaro M, Pisano G et al (2020) Virtual surgical planning for reconstruction of giant ameloblastoma of the mandible. Ann Plast Surg 85(1):43–49 32530830 10.1097/SAP.0000000000002390

[19] John B, Wickramasinghe N. (2020) A review of mixed reality in health care. In Healthcare delivery in the information age: delivering superior health and wellness management with IoT ad analytics. Springer.

[20] Kouijzer MMTE, Kip H, Bouman YHA, Kelders SM. (2023) Implementation of virtual reality in healthcare: a scoping review on the implementation process of virtual reality in various healthcare settings. Implement Sci Commun 4(1). 10.1186/s43058-023-00442-2 PMC10276472 37328858

[21] Page MJ, McKenzie JE, Bossuyt PM, Boutron I, Hoffmann TC, Mulrow CD, et al. (2020). The PRISMA 2020 statement: an updated guideline for reporting systematic reviews. The BMJ 372. BMJ Publishing Group. 10.1136/bmj.n71 PMC8005924 33782057

[22] Zinser MJ, Mischkowski RA, Dreiseidler T, Thamm OC, Rothamel D, Zöller JE (2013) Computer-assisted orthognathic surgery: waferless maxillary positioning, versatility, and accuracy of an image-guided visualisation display. Br J Oral Maxillofac Surg 51(8):827–833 24045105 10.1016/j.bjoms.2013.06.014

[23] Badiali G, Roncari A, Bianchi A, Taddei F, Marchetti C, Schileo E (2015) Navigation in orthognathic surgery: 3D accuracy. Facial Plast Surg 31(5):463–473 26579862 10.1055/s-0035-1564716

[24] Lutz JC, Nicolau S, Agnus V, Bodin F, Wilk A, Bruant-Rodier C, Rémond Y, Soler L (2015) A novel navigation system for maxillary positioning in orthognathic surgery: preclinical evaluation. J Cranio-Maxillofac Surg 43(9):1723–1730 10.1016/j.jcms.2015.08.001 26364761

[25] Steinhuber T, Brunold S, Gärtner C, Offermanns V, Ulmer H, Ploder O (2018) Is virtual surgical planning in orthognathic surgery faster than conventional planning? A time and workflow analysis of an office-based workflow for single- and double-jaw surgery. J Oral Maxillofac Surg 76(2):397–407 28826783 10.1016/j.joms.2017.07.162

[26] Gao Y, Lin L, Chai G, Xie L (2019) A feasibility study of a new method to enhance the augmented reality navigation effect in mandibular angle split osteotomy. J Cranio-Maxillofac Surg 47(8):1242–1248 10.1016/j.jcms.2019.04.005 31080053

[27] Zaragoza-Siqueiros J, Medellin-Castillo HI, Garza-Camargo H, Lim T, Ritchie JM (2019) An integrated haptic-enabled virtual reality system for orthognathic surgery planning. Comput Methods Biomech Biomed Engin 22(5):499–517 30714408 10.1080/10255842.2019.1566817

[28] Medellin Castillo HI, Zaragoza-Siqueiros J, Govea-Valladares EH, de la Garza-Camargo H, Lim T, Ritchie JM (2021) Haptic-enabled virtual training in orthognathic surgery. Virtual Real 25(1):53–67 10.1007/s10055-020-00438-6

[29] Ricciardi F, Copelli C, De Paolis LT. (2017) An augmented reality system for maxillo-facial surgery. Springer International Publishing: 53–62.

[30] Wu W, Chen H, Cen Y, Hong Y, Khambay B, Heng PA (2017) Haptic simulation framework for determining virtual dental occlusion. Int J Comput Assist Radiol Surg 12(4):595–606 27601232 10.1007/s11548-016-1475-3

[31] Ahn J, Choi H, Hong J, Hong J (2019) Tracking accuracy of a stereo camera-based augmented reality navigation system for orthognathic surgery. J Oral Maxillofac Surg 77(5):1070.e1-1070.e11 30707984 10.1016/j.joms.2018.12.032

[32] Koyachi M, Sugahara K, Odaka K, Matsunaga S, Abe S, Sugimoto M, Katakura A (2021) Accuracy of Le Fort I osteotomy with combined computer-aided design/computer-aided manufacturing technology and mixed reality. Int J Oral Maxillofac Surg 50(6):782–790 33158695 10.1016/j.ijom.2020.09.026

[33] Jo YJ, Choi JS, Kim J, Kim HJ, Moon SY. (2021) Virtual reality (VR) simulation and augmented reality (AR) navigation in orthognathic surgery: a case report. Applied Sciences (Switzerland) 11(12).

[34] Pulijala Y, Ma M, Pears M, Peebles D, Ayoub A (2018) An innovative virtual reality training tool for orthognathic surgery. Int J Oral Maxillofac Surg 47(9):1199–1205 29398172 10.1016/j.ijom.2018.01.005

[35] Arikatla V, Tyagi M, Enquobahrie A, Nguyen T, Blakey GH, White R, Paniagua B. (2018) High fidelity virtual reality orthognathic surgery simulator. Proc SPIE Int Soc Opt Eng. 10.1117/12.2293690 PMC6028926 29977103

[36] Pham Dang N, Chandelon K, Barthélémy I, Devoize L, Bartoli A (2021) A proof-of-concept augmented reality system in oral and maxillofacial surgery. J Stomatol Oral Maxillofac Surg 122(4):338–342 34087435 10.1016/j.jormas.2021.05.012

[37] Moola S, Munn Z, Tufanaru C, Aromataris E, Sears K, Sfetcu R, et al. (2017). Checklist for analytical cross sectional studies critical appraisal checklist for analytical cross-sectional studies 2. http://joannabriggs.org/research/critical-appraisal-tools.htmlwww.joannabriggs.org

[38] Moola S, Munn Z, Tufanaru C, Aromataris E, Sears K, Sfetcu R, et al. (2020). JBI critical appraisal checklist for case reports. Chapter 7: systematic reviews of etiology and risk.

[39] Tufanaru C, Munn Z, Aromataris E, Campbell J, Hopp L. (2020) JBI critical appraisal checklist for randomized controlled trials. Chapter 3: systematic reviews of effectiveness. https://synthesismanual.jbi.global

[40] Lutz JC, Hostettler A, AgnusV NS, George D, Soler L, Rémond Y (2019) A new software suite in orthognathic surgery : patient specific modeling, simulation and navigation. Surg Innov 26(1):5–20 30270757 10.1177/1553350618803233

[41] Steinhuber T, Brunold S, Gärtner C, Offermanns V, Ulmer H, Polder O (2017) Is virtual surgical planning in orthognathic surgery faster than conventional planning? A time and workflow analysis of an office-based workflow for single- and double-jaw surgery. J Oral Maxillofac Surg 76(2):397–407 28826783 10.1016/j.joms.2017.07.162

[42] Kim MG, Ryu JS, Son J, Han JH (2022) Virtual object sizes for efficient and convenient mid-air manipulation. Visual Computer 38(9–10):3463–3474 35791413 10.1007/s00371-022-02555-6 PMC9247950

[43] Pulijala Y, Ma M, Pears M, Peebles D, Ayoub A (2018) Effectiveness of immersive virtual reality in surgical training-a randomized control trial. J Oral Maxillofac Surg 76(5):1065–1072 29104028 10.1016/j.joms.2017.10.002

[44] Bhugaonkar Km Bhugaonkar R, Masne N. (2022) The trend of metaverse and augmented & virtual reality extending to the healthcare system. Cureus. 10.7759/cureus.29071 PMC9559180 36258985

[45] Bengtsson M, Wall G, Becktor JP, Rasmusson L (2019) A comparison of cost-effectiveness of computer-assisted 2-and 3-dimensional planning techniques in orthognathic surgery. Br J Oral Maxillofac Surg 57(4):352–358 30962030 10.1016/j.bjoms.2019.03.012

[46] Abbate V, Committeri U, Troise S, Bonavolontà P, Vaira LA, Gabriele G, Biglioli F, Tarabbia F, Califano L, Dell’Aversana Orabona G. (2023). Virtual surgical reduction in atrophic edentulous mandible fractures: a novel approach based on “in house” digital work-flow. Applied Sci (Switzerland), 13(3).

[47] Resnick CM, Inverso G, Wrzosek M, Padwa BL, Kaban LB, Peacock ZS (2016) Is there a difference in cost between standard and virtual surgical planning for orthognathic surgery? J Oral Maxillofac Surg 74(9):1827–1833 27181623 10.1016/j.joms.2016.03.035

[48] Mccloy R, Stone R (2001) Clinical review Science, medicine, and the future: virtual reality in surgery. Manchester BMJ 323:912–915 11668138 10.1136/bmj.323.7318.912 PMC1121442

[49] Sutherland J, Belec J, Sheikh A, Chepelev L, Althobaity W, Chow BJW, Mitsouras D, Christensen, A, Rybicki FJ, la Russa DJ. (2019). Applying modern virtual and augmented reality technologies to medical images and models. In Journal of digital imaging (Vol. 32, Issue 1, pp. 38–53). Springer New York LLC. 10.1007/s10278-018-0122-7 10.1007/s10278-018-0122-7 PMC6382635 30215180

[50] Koyachi M, Sugahara K, Tachizawa K, Nishiyama A, Odaka K, Matsunaga S, Sugimoto M, Tachiki C, Nishii Y, Katakura A. (2023). Enhanced precision in genioplasty: a novel intraoperative spatial repositioning using computer-aided design and manufacturing technology and a holographic mixed reality application. J Clin Med, 12(23). 10.3390/jcm12237408 PMC10707674 38068460

[51] Chen Z, Mo S, Fan X, You Y, Ye G, Zhou N. (2021) A meta-analysis and systematic review comparing the effectiveness of traditional and virtual surgical planning for orthognathic surgery: based on randomized clinical trials. J Oral Maxillofac Surg 79(2). W.B. Saunders: 471.e1–471.e19. 10.1016/j.joms.2020.09.005 33031773

[52] Murdoch B. (2021) Privacy and artificial intelligence: challenges for protecting health information in a new era. BMC Med Ethics 22(1). 10.1186/s12910-021-00687-3 PMC8442400 34525993

[53] Lim YY, Poronnik P, Usherwood T, Reeve B (2020) Medical negligence laws and virtual reality in healthcare. Aust J Gen Pract 49(8):525–529 32738869 10.31128/AJGP-08-19-5036

[54] US Food & Drug Administration. (2022) Executive summary for the patient engagement advisory committee meeting. In Augmented reality and virtual reality medical devices, https://www.fda.gov

